# Functions and Epigenetic Regulation of Wwox in Bone Metastasis from Breast Carcinoma: Comparison with Primary Tumors

**DOI:** 10.3390/ijms18010075

**Published:** 2017-01-01

**Authors:** Paola Maroni, Emanuela Matteucci, Paola Bendinelli, Maria Alfonsina Desiderio

**Affiliations:** 1 Galeazzi Orthopaedic Institute, Scientific Institute for Research, Hospitalization and Health Care (IRCCS), 20161 Milano, Italy; paola.maroni@grupposandonato.it; 2 Department of Biomedical Sciences for Health, Molecular Pathology Laboratory, Università degli Studi di Milano, 20133 Milano, Italy; emanuela.matteucci@unimi.it (E.M.); paola.bendinelli@unimi.it (P.B.)

**Keywords:** bone metastasis, epigenetic reprogramming, Wwox, transcription factors

## Abstract

Epigenetic mechanisms influence molecular patterns important for the bone-metastatic process, and here we highlight the role of WW-domain containing oxidoreductase (Wwox). The tumor-suppressor Wwox lacks in almost all cancer types; the variable expression in osteosarcomas is related to lung-metastasis formation, and exogenous Wwox destabilizes HIF-1α (subunit of Hypoxia inducible Factor-1, HIF-1) affecting aerobic glycolysis. Our recent studies show critical functions of Wwox present in 1833-osteotropic clone, in the corresponding xenograft model, and in human bone metastasis from breast carcinoma. In hypoxic-bone metastatic cells, Wwox enhances HIF-1α stabilization, phosphorylation, and nuclear translocation. Consistently, in bone-metastasis specimens Wwox localizes in cytosolic/perinuclear area, while TAZ (transcriptional co-activator with PDZ-binding motif) and HIF-1α co-localize in nuclei, playing specific regulatory mechanisms: TAZ is a co-factor of HIF-1, and Wwox regulates HIF-1 activity by controlling HIF-1α. In vitro, DNA methylation affects Wwox-protein synthesis; hypoxia decreases Wwox-protein level; hepatocyte growth factor (HGF) phosphorylates Wwox driving its nuclear shuttle, and counteracting a Twist program important for the epithelial phenotype and metastasis colonization. In agreement, in 1833-xenograft mice under DNA-methyltransferase blockade with decitabine, Wwox increases in nuclei/cytosol counteracting bone metastasis with prolongation of the survival. However, Wwox seems relevant for the autophagic process which sustains metastasis, enhancing more Beclin-1 than p62 protein levels, and p62 accumulates under decitabine consistent with adaptability of metastasis to therapy. In conclusion, Wwox methylation as a bone-metastasis therapeutic target would depend on autophagy conditions, and epigenetic mechanisms regulating Wwox may influence the phenotype of bone metastasis.

## 1. Introduction

The formation of metastasis is a complex process, and the molecular events involved and their genetic and epigenetic regulation vary depending on the primary tumor and the organotropism. Recent studies deal with the role of proteins containing the WW structural module, critical for protein-protein interaction, which play a multitude of functions through signaling networks. It is intriguing the interaction of the co-factor Wwox (WW-domain containing oxidoreductase) with Hippo pathway, which promotes cell death and differentiation counteracting cell proliferation [1]. Surprisingly, Hippo-signaling pathway seems also involved in tumorigenesis and metastasis in relation to the different patterns of Wwox [2,3]. In view of the plastic phenotype of metastases, and the leading role played by epigenetic mechanisms, in the present review we dedicate a section to the methylation-dependent functions of the tumor-suppressor Wwox, which is expressed in bone metastases from breast carcinoma. Our newest observations are also shown and discussed to give a better idea of the significance of Wwox in the autophagy process occurring in bone metastases, and to update the knowledge of the cellular and molecular events triggered by DNA methyltransferase blockade. Some of these points are highlighted taking into consideration that epigenetic reprogramming is important for metastatic adaptability to the different microenvironments encountered during the various steps from dissemination until bone colonization [4].

## 2. Lack of *WWOX* Gene Expression in Tumors, and Functional Effects of Exogenous Wwox

**Figure 1:**
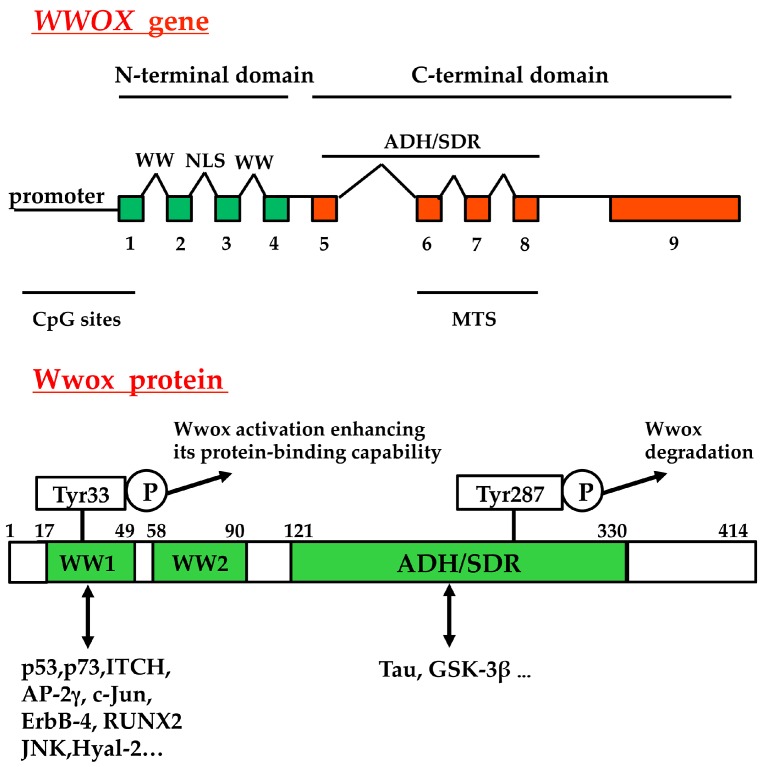
Structure of *WWOX* gene and of the mature protein. The nine exons of the gene, and the domains for protein-protein interactions are indicated. WW, WW domain; NLS, nuclear localization sequence; ADH/SDR, short-chain alcohol dehydrogenase/reductase; MTS, mitochondrial targeting sequence; CpG sites, methylation sites; ITCH, E3 ubiquitin ligase; Runx2, Runt-related transcription factor 2; Hyal-2, hyaluronidase-2; GSK-3β, glycogen synthase kinase 3β. P, phospho. For Wwox protein, the amino acid position is indicated by the numbers.

The human *WWOX* gene, encoding a 46 kDa protein, localizes on the chromosome 16q23.3–24.1 and consists of nine exons; at least eight alternative spliced mRNAs are known [5,6,7,8,9,10,11]. Putative methylation sequences occur in the *WWOX* gene: 62 CpG sites span the *WWOX* promoter, exon 1, and a small region of intron 1 [12]. As shown in Figure 1, in the full-length Wwox two WW domains are present in the N-terminal region, and a nuclear localization signal is located between the first and second WW domain. A short-chain alcohol dehydrogenase/reductase (ADH/SDR) is present in the C-terminal domain, where a mitochondria-targeting sequence is mapped [13]. The domains for the interaction of co-factors, which regulate Wwox activity, and the phosphorylation sites are indicated.

Wwox is involved in various patho-physiological conditions such as neural development, neural damage and neurodegeneration [13,14,15,16,17,18], bone metabolism [19,20], and osteoarthritis [21].

In agreement with Wwox function as tumor suppressor, *WWOX* gene spans a common fragile site FRA16D [5,6,7,8,9,10,11]. FRA16D, identified as one of the various sites of small homozygous deletions in cancer [22], belongs to the almost 200 fragile sites corresponding to regions of DNA instability in different types of cancer. The perturbation of the function of the genes with common fragile sites suggests their role in cancer cell biology [23]. *WWOX* does not seem to act as a highly penetrant classical tumor suppressor [24], and low levels of Wwox, rather than complete absence of Wwox, are often observed in tumors, indicating that the tumor suppressor function of Wwox does not fit Knudson’s two hit hypothesis of tumorigenesis. Most intriguingly, protein levels of Wwox are remarkably increased in the early stages of hyperplasia and cancerous progression of breast and prostate cancer [25,26], and the significance might be that Wwox arrests cancer growth in the preneoplastic stages, even if other scenarios cannot be excluded.

Reduced levels of full-length Wwox protein have been reported in multiple types of cancer [11,27,28,29]. In particular, Wwox reduction or loss is observed in human osteosarcoma, but Wwox expression is related to osteosarcoma aggressiveness and formation of lung metastases. Osteosarcoma development seems to depend on destruction of the Wwox-p53 network in osteoprogenitors [30].

Evidence of individuals with low Wwox-protein levels, being more predisposed to the development of lung cancer and gliomas, also supports a role for *WWOX* in tumorigenesis [31,32]. Aberrant *WWOX* transcripts are often observed in tumors with reduced levels of full-length Wwox [27,33,34,35]. These transcripts mostly lack part, if not all, of the exons that encode its SDR enzyme, and several of these transcripts are translated into truncated protein products lacking the complete SDR enzyme, supporting oxidative stress [34,35].

Inactivation of *WWOX* gene participates in the progression of head and neck squamous cell carcinoma (HNSCC). The downregulation of *WWOX* expression in advanced-stage tumor samples of HNSCC is associated with methylation of the *WWOX* promoter region but not with miR-134 expression. The expression level of *WWOX* and the methylation of the promoter are inversely correlated: 10 different alterations in the coding sequences and 18 different alterations in the non-coding sequences of the *WWOX* gene in HNSCC tumor samples are reported. Thus, *WWOX* gene can be inactivated epigenetically by promoter methylation, or functionally by mutations affecting the sequences coding for the enzymatic domain of the gene [36].

Altered metabolism is now recognized as one of the hallmarks of cancer cells, due to mutations of enzymes of the tricarboxylic acid cycle and of the mitochondrial respiratory chain [37]. A novel functional contribution of Wwox in moderating the mitochondria-mediated pathway, related to the aerobic metabolism, depends upon its SDR enzyme function [38].

**Figure 2:**
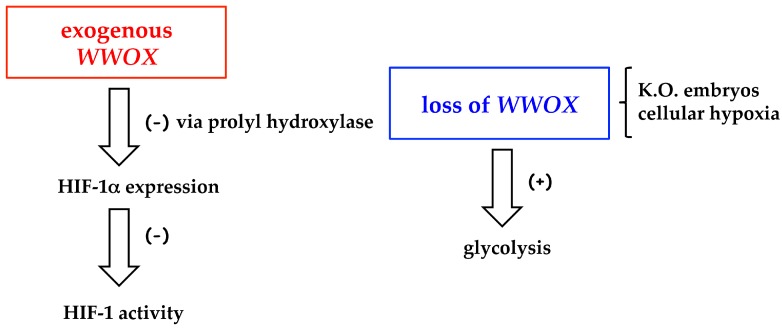
Regulation of HIF-1 (Hypoxia inducible factor-1) activity and glycolysis by exogenous *WWOX* and *WWOX* loss. K.O., knockout.

Moreover, the loss of Wwox seems to contribute to the metabolic reprogramming of cells that accompanies tumorigenesis (Figure 2).

Hypoxia inducible factor-1 (HIF-1) is the master regulator of oxygen homeostasis and of aerobic glycolysis in tumors, and HIF-1α is the inducible subunit [39]. As shown by Abu-Remaileh and Aqeilan, exogenous Wwox decreases HIF-1α protein level both in vitro and in vivo; *WWOX* knockout mice exhibit enhanced levels of serum lactic acid [20]. The mechanisms proposed are the degradation of HIF-1α via prolyl hydroxylase control, and the suppression of HIF-1 transcriptional function [20]. The loss of *WWOX* is responsible for the activation of the glycolysis in mouse embryonic fibroblasts (MEF) from knockout (K.O.) embryos, compared with fibroblasts from *WWOX*-wild type embryos [40]. Notwithstanding hypoxia downregulates Wwox in different cell types as in 1833-bone metastatic cells, derived from MDA-MB231 breast carcinoma cells [20,41], only in 1833 cells exogenous Wwox stabilizes HIF-1α: the latter effect is more pronounced under moderate hypoxia. The underlying mechanism has been studied by us [41] (see the paragraph on Wwox in bone metastasis), and might explain some aspects of the control of HIF-1-dependent gene pattern in skeleton metastasis expressing Wwox.

In breast carcinoma cells, Wwox antagonizes the function of YAP (Yes-associated protein), regulating in nuclei the transcriptional activity of tyrosine kinase receptors; overexpression of Wwox suppresses the activity of the transcription factors p73, AP2, Erb4, c-Jun, and Met, sequestering them in the cytosol [42,43]. YAP is a WW-domain containing regulatory protein, and we deepen the significance using two breast carcinoma cell lines (i.e., the invasive MDA-MB231 and the non-invasive MCF-7 cells). Only in MDA-MB231 cells, Met receptor fragments (Met-CTF) are present in nuclei, and they play a transactivating function: in these cells Wwox is lacking, and in nuclei YAP- phosphorylated by Akt- intervenes as a co-factor in Met-CTF activity important for spontaneous invasiveness. Exogenous Wwox overexpression causes Met loss of function, due to the impairment of Met-CTF nuclear localization [42].

Other functions of Wwox are related to signal transduction pathways. In colon carcinoma cells HCT116 deficient in TGFβRII, TGFβ1 binds cell surface hyaluronidase Hyal-2, followed by a rapid recruitment of Wwox. The resulting Wwox/Hyal-2 complex recruits Smad4, relocates to the nuclei enhancing Smad promoter activation, important for the control of gene transcription [2]. Ezrin, a member of the membrane-cytoskeleton linker family, is implicated in a variety of cellular dynamics such as determination of cell shape, adhesion, motility, and survival; ezrin interacts with Wwox determining its apical localization, which is important for proton pump H,K-ATPase recruitment to apical membrane during the gastric-parietal cell activation [44].

## 3. The Hippo Pathway in Tumorigenesis

### 3.1. YAP and TAZ Function as Transcriptional Co-Activators

YAP and TAZ (transcriptional co-activator with PDZ-binding motif, and one WW domain) are proteins of the core of Hippo pathway, and regulate gene transcription critical for embryonic development and tumorigenesis by signal transduction from the cytoplasm to the nucleus [45]. YAP and TAZ do not contain intrinsic DNA-binding domains, but they may interact with transcription factors targeting specific gene promoters [3]. A further complexity is given by the inhibition of these core proteins downstream of the Hippo pathway via LATS1/2 phosphorylation, activated by MST1/2 [46,47].

YAP1 contributes to the KRas-dependent transformed phenotype, and it drives proliferation by acting as cofactor for TEA Domain (TEAD) transcriptional regulator; when TEAD association is disrupted, YAP1 activity is lost [48]. The ablation of epithelial integrity, together with subsequent YAP1 nuclear localization, allows transcriptional activation of β-catenin/TEX-YAP/TEAD target genes, including Myc, responsible for the invasive phenotype [49].

### 3.2. Aberrant Signaling in Tumors

In model systems, genetic ablation of core Hippo pathway components leads to increased tumorigenesis [48]. Published data on humans report either mutations of Guanine nucleotide-binding protein Gq subunit α (GNAQ), as in uveal melanoma, or inactivation of NF2/merlin, as in the tumor-prone neurofibromatosis syndrome. These mutations may hamper the inhibitory signal of LATS1 permitting YAP1 activation: the latter occurs independently of Hippo kinase MST itself. Similarly, molecular mechanisms regulated by stroma and genetic instability are triggered independent of LATS1/2 phosphorylation activated by MST1/2 [48].

Proteomic studies identify that the main direct activators of MST kinases are SAV1 and RASSFs [50]. Although infrequently mutated in cancers [51], germline and epigenetic alterations involve particularly RASSF1A, accelerating tumor onset and increasing tumorigenicity [52,53]. Distinct RASSF1 isoforms have opposite functions, providing biomarkers for YAP1 activation, and explaining the correlations of RASSF1 methylation with invasive growth in humans.

Interesting advancement regards the identification of numerous upstream components of the Hippo pathway, such as cell polarity, mechanotransduction, and G-protein coupled receptor signaling. However, in tumors, the effect of deranged upstream components on Hippo pathway function is scarcely known. Physical attachment of normal cells to extracellular matrix is essential for survival and growth, due to the YAP nuclear localization, and the activation of Rho-GTPases or of the FAK-Src-PI3K pathway [54,55]. Rho-GTPases and the actin cytoskeleton play an essential role in the regulation of YAP and TAZ by mechanotransduction. Tensive forces couple to the cytoskeleton to integrate and transmit upstream signals to the core Hippo signaling cascade [1], and the mechanical strain suppresses YAP phosphorylation promoting YAP nuclear translocation by inactivating LATS1/2 in a JNK-dependent manner [56]. Thus, the Hippo pathway not only intervenes in the growth inhibitory signal due to cell-cell contact at high cell density [47], but it also permits to overcome the physical restrictions due to extracellular mechanical cues, favoring growth and development. Nuclear YAP or TAZ seems to influence TGFβ signaling towards an aggressive phenotype. In fact, YAP/TAZ may hamper tumor-suppressive events, recruiting NCoR/HDAC3 chromatin-remodeling complex to gene promoters [3].

Exciting findings indicate that epigenetic mechanisms might deregulate the tumor suppressive Hippo pathway and the signaling downstream implicated in tumorigenesis.

## 4. Wwox Influences the Gene Pattern of Human Bone Metastasis from Breast Carcinoma

**Figure 3:**
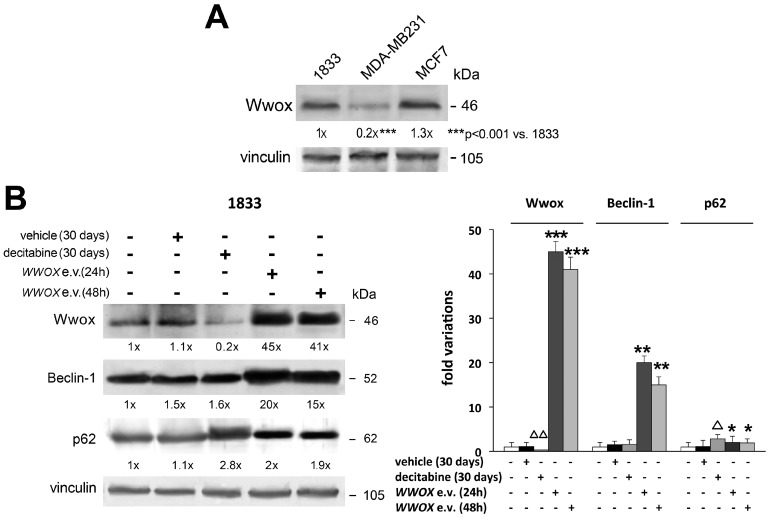
Wwox protein levels in breast carcinoma cell lines, and role of decitabine and Wwox in the expression of autophagy players. All Western blots were performed with 100 µg of total proteins, and the specific signals were examined after immunoblotting and reaction with ECL plus chemiluminescence kit. Vinculin was used for normalization of the densitometric values. (**A**) Wwox protein levels were evaluated in different cell lines: bone metastatic 1833 cells, derived from invasive MDA-MB231 breast carcinoma cells; MCF-7 non-invasive breast carcinoma cells; (**B**) the 1833 cells were exposed to decitabine or the vehicle alone for 30 days; *WWOX* expression vector (e.v.) was transfected for 24 or 48 h [64]. The first lanes correspond to control-untreated cells. After normalization of the densitometric values using vinculin, the data were used for the graphic. The Western blot experiments in (**A**,**B**) were repeated three times, and the data show the fold variations versus the first lane. The statistical analysis was performed using ANOVA, with *p* < 0.05 considered significant. * *p* < 0.05, ** *p* < 0.005, *** *p* < 0.001 versus control; ^Δ^ *p* < 0.05, ^ΔΔ^ *p* < 0.005 versus vehicle.

Recently, we reported unexpected findings that Wwox tumor suppressor is elevated in specimens of human bone metastasis. While being almost absent in primary invasive ductal breast carcinoma, the adjacent mammary tissue shows Wwox signal. In human bone metastasis, Wwox localizes at cytosolic-perinuclear and at nuclear levels [41,57,58]. Consistently, endogenous Wwox expression is remarkably higher in 1833 bone-metastatic clone than in invasive MDA-MB231 breast carcinoma cells, and it is similarly elevated in non-invasive MCF-7 breast carcinoma cells (Figure 3A).

These data stimulate the studies of Wwox function and regulation in bone metastases. Recent findings indicate the association of *WWOX* gene polymorphism with the risk of metastasis from osteosarcoma, that is known to metastasize in the brain and lungs [59]. In any case, Wwox expression and function in metastases other than the osseous one are practically unknown due to the difficulties to obtain human samples and to prepare animal models.

The adaptability of the disseminated cancer cells to the different microenvironments encountered during the various steps of the metastatic process depends on the dynamic phenotype, which is regulated principally by epigenetic mechanisms and hypoxia [39,60,61,62]. Importantly, in 1833 cells Wwox expression varies with DNA-methylation state and hypoxic conditions [41,57]. These points deserve discussion, and clarify the significance of Wwox in bone metastases.

First, the endogenous level of Wwox protein in 1833 cells is regulated by DNA methyltransferases, and long-term exposure to the inhibitor 5-azacytidine causes Wwox downregulation [57]. The 5-azacytidine molecule is incorporated into newly synthesized DNA after conversion to 5-aza-2′-deoxycytidine [63]. Of note, the treatment of 1833-xenograft mice with the inhibitor of DNA methyltransferases 5-aza-2′-deoxycytidine (decitabine), prolongs mouse survival and increases Wwox expression both in cytosol and nuclei. Oppositely, hepatocyte growth factor (HGF)/Met receptor, one of the principal bone-metastatic regulatory axis, is downregulated [64]. An explanation might be that microenvironmental stimuli of bone metastases, such as HGF, influence Wwox level, phosphorylation and function. HGF controls a Twist program for transient mesenchymal-epithelial transition, which is important for colonization, via *E*-cadherin expression and vimentin/MMP2 downregulation, and probably also for osteoclast activity [58]. HGF permits Twist access into the nuclei in a phosphorylated form, while Wwox phosphorylated exchanges with Twist, which shuttles in the cytosol in unphosphorylated form ending the program. A TGFβ1 signaling is activated downstream HGF, contributing to the plastic phenotype of bone metastasis and to the colonization associated with osteolysis [58].

Altogether, to devise therapies targeting the epithelial phenotype, or the reverse mesenchymal phenotype in a time-dependent manner, the situation of biological and physical (hypoxia) stimuli of the microenvironment has to be considered, because they might co-operate influencing Wwox function and also the efficacy of DNA methyltransferase blockade.

Secondly, to clarify the significance of elevated Wwox expression in bone metastasis, Maroni et al. reproduce this condition through the ectopic expression of Wwox [41]. In 1833 and MDA-MB231 cells transfected with *WWOX*-expression vector, invasiveness through Matrigel increases and decreases, respectively, even if Wwox protein accumulates in both the cell lines: this finding indicates different effects of exogenous Wwox on the gene patterns for the motile phenotype depending on the cell type [57]. After enhancement of Wwox-mediated migration through Matrigel, the 1833 cells undertake contacts consistent with *E*-cadherin expression [57]. A key mechanism for modulating cell-cell adhesion strength is the adjustment of the amount of *E*-cadherin [65], and Wwox influencing *E*-cadherin expression contributes to mesenchymal-epithelial transition and colonization of bone metastasis from breast carcinoma [58].

The re-expression of *E*-cadherin in bone metastasis contributes to aberrant homotypic/heterotypic cell adhesion, motility and transduction signaling [66], and Wwox is likely to influence *E*-cadherin intracellular localization and function. *E*-cadherin-containing surface structures affect adhesion and colonization of bone metastasis [67]: redistribution of COOH-*E*-cadherin fragment in nuclei seems to favor invasion [68]. Notably, in humans *E*-cadherin and Wwox are expressed in bone metastasis but not in breast carcinoma, while HIF-1α and TAZ are localized principally in nuclei of metastasis and throughout breast carcinoma cells [41]: TAZ is a HIF-1 co-factor in bone metastases [69].

Importantly, we show a molecular mechanism typical of skeletal metastasis, which implicates Wwox as a player in the HIF-1α-HDM2 regulatory loop, necessary for the dynamic regulation of HIF-1α amount and HIF-1 activity. Hypoxia is additive with Wwox, remarkably enhancing HIF-1α nuclear shuttle and accumulation due to lengthening of the half-life of HIF-1α protein. By interacting with HDM2 and sequestering the E3-ubiquitin ligase, Wwox is likely to prevent HIF-1α degradation under mild hypoxia favoring HIF-1 activity [41]. The effect of exogenous Wwox on the metabolism of metastatic cells via aerobic glycolysis has not been investigated. In primary breast carcinoma with low or null Wwox, HIF-1α levels are controlled through stabilization dependent on hypoxia and oncogenes [39].

In contrast to oxidative stress and energy stress, hypoxia causes YAP and TAZ activation by inhibiting LATS through the SIAH2-E3 ubiquitin ligase, which destabilizes LATS2 [47]. In 1833 clone, but not in parental MDA-MB231 cells, hypoxia induces *E*-cadherin through HIF-1 and PPARγ activities [41].

Thirdly, the intracellular localization of Wwox is important for the function, and may vary depending upon the status of cell differentiation and the types of cells tested [6]. In normal keratinocytes Wwox frequently localizes in the perinuclear area, and when they start undergoing cornification it accumulates in the nucleus [6]. Numerous biological and stress stimuli induce re-location of Wwox to the nuclei. In response to TNF-α, TGF-β, staurosporine, etoposide, ultraviolet irradiation, complement C1q, and sex steroid hormones (androgen and estrogen), Wwox phosphorylation at Tyr33 occurs in many cultured cell lines: phosphorylated or activated Wwox translocates to the mitochondria and nuclei [25,70,71,72,73].

Finally, Wwox seems implicated in autophagy in agreement with the pro-metastatic function of the autophagic process, which instead would inhibit tumorigenesis, even if this remains still controversial [74]. The molecular mechanisms underlying the various steps of autophagy are not completely clarified. Two key players of the autophagy are Beclin-1 index of the autophagic flux, being involved in isolation/membrane initiation, and p62-scaffold protein, which may be substrate of autophagy or adapter regulating survival pathways involving NF-κB activity [75,76]. As shown in Figure 3B, the transfection of *WWOX* expression vector increased strongly Beclin-1 and less p62 protein levels in 1833 cells (quantification of the data is included in the graphic). Ectopic Wwox, that in vivo might derive from microenvironment supportive cells of bone metastasis, is likely to influence the initial autophagosome formation and the following autolysosome function where Beclin-1 and p62 are critically implicated. One of the two events, i.e., autophagy activation or block of p62 degradation, might be sufficient to cope with the environment or cell stress conditions in bone metastasis. It can be hypothesized that Wwox per se influences p62 induction notwithstanding autophagy flux: p62 is affected by oxidative stress and Wwox seems to cause the formation of reactive oxygen species [38,76]. Under decitabine, the p62 degradation was prevented probably due to the impairment of the autophagic process being Beclin-1 unchanged and Wwox downregulated. As a consequence of autophagy failure, the metastatic cells are under stressful conditions which induced p62, because of the hampering of energy supply and removal of damaged organelles. Further studies are needed to clarify the significance of p62 in bone metastasis under therapy targeting DNA methyltransferases.

The data on autophagy contribute to clarifying the effect of decitabine in mice bearing bone metastases from breast carcinoma. The efficacy of decitabine, a chemotherapic drug for various kinds of primary tumors never tested to fight bone metastases, might be strongly influenced by the microenvironment biological stimuli acting on the molecular mediators like Wwox, and by the autophagy conditions.

## 5. Conclusions

**Figure 4:**
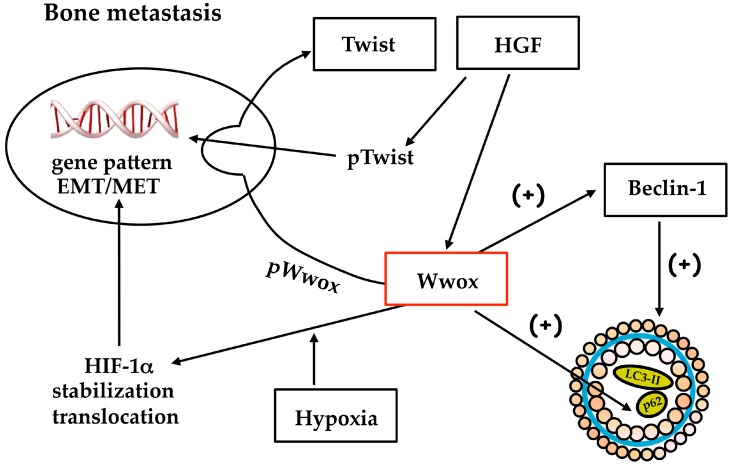
Wwox plays various functions in bone metastasis, related to gene expression for mesenchymal epithelial transition and autophagy. EMT, epithelial-mesenchymal transition; MET, mesenchymal-epithelial transition; HGF, hepatocyte growth factor.

Wwox’s role in cancer progression is one of the intensely debated concepts, because of its double nature in primary tumor growth and metastatic process, and its relation with HIF-1α expression/HIF-1 activity. Wwox may influence the gene pattern underlying the phenotype of bone metastasis: Wwox counteracts YAP function, positive for HGF/Met receptor signaling, and activates HIF-1 with upregulation of *E*-cadherin; finally, phosphoWwox downregulates Twist transactivation, reverting the epithelial phenotype important for colonization. Altogether, Wwox seems to play key functions in bone metastasis from breast carcinoma (Figure 4).

Wwox drives the switch towards an epithelial phenotype due to *E*-cadherin expression, critical for osteoblastic niche formation and colonization, and the autophagy triggering which might be implicated in survival of metastatic cells engrafted in the bone. We suggest the importance for metastasis therapy of Wwox involvement in the nuclear shift (i) of HIF-1α subunit-contributing to HIF-1 activation under hypoxia; and (ii) of Twist under HGF: the phosphorylation and stabilization of these transcription factors lead to the expression of genes critical for the bone-metastatic process. The p62 saved from degradation because of Wwox-dependent interference in the autophagosome sealing and lipidation with LC3II, might activate NF-κB. The latter transcription factor controls a gene pattern (HIF-1α, COX2, ornithine decarboxylase) important for metastasis growth [41,77,78] and apoptosis resistance [79]. Wwox is regulated by methylation, and the chemotherapic drug decitabine might be useful to affect bone metastasis colonization, preventing Met receptor fragment and Twist access to nuclei, but the efficacy might dependent on autophagy conditions.

## Abbreviations

YAP	Yes-associated protein
TAZ	Transcriptional co-activator with PDZ-binding motif
Wwox	WW-domain containing oxidoreductase
TEAD	TEA Domain
ADH/SDR	Short-chain alcohol dehydrogenase/reductase
HIF-1	Hypoxia inducible factor-1
Met-CTF	Met receptor fragments
MET	Mesenchymal-epithelial transition
HGF	Hepatocyte growth factor
